# Galectin-1 as a predictive biomarker in ovarian cancer

**DOI:** 10.1186/s13048-021-00874-1

**Published:** 2021-09-23

**Authors:** Mahak Masoodi, Zafar A. Shah, Afaq H. Beigh, Sheikh Zahoor Ahmad, Abdul Wahid Mir, Besina Yasin, Roohi Rasool, Khalid Z. Masoodi, Gull Mohammad Bhat

**Affiliations:** 1 Department of Immunology and Molecular Medicine, Sher-e-Kashmir Institute of Medical Sciences, Srinagar, Jammu and Kashmir India; 2 Department of Surgical Oncology, Sher-e-Kashmir Institute of Medical Sciences, Srinagar, Jammu and Kashmir India; 3 Department of Pathology, Sher-e-Kashmir Institute of Medical Sciences, Srinagar, Jammu and Kashmir India; 4 Division of Plant Biotechnology, Sher-e-Kashmir University of Agricultural Sciences and Technology, Srinagar, Jammu and Kashmir India; 5 Department of Medical Oncology, Sher-e-Kashmir Institute of Medical Sciences, Srinagar, Jammu and Kashmir India

**Keywords:** Galectin-1, Ovarian Cancer, CA125, Biomarker, ELISA

## Abstract

**Aim:**

There is an urgent need to set up a useful biomarker for ovarian cancer. Galectin-1 is a promising carbohydrate-binding protein which plays a remarkable role in various malignancies yet its clinical significance is questionable. In this study, we have tested the clinical implications of serum Galectin-1 levels in patients with ovarian tumours.

**Main methods:**

Serum Galectin-1 levels were quantified in 84 newly diagnosed ovarian tumour patients and 20 healthy controls by Enzyme Linked Immuno Sorbent Assay during the course of the disease. Therefore the samples were taken at diagnosis, after surgery and after chemotherapy.

**Key findings:**

The Galectin-1 levels were found to be associated with various variables of Ovarian Cancer patients. The levels were found to be prominently high in postmenopausal patients. Galectin-1 levels were raised in epithelial ovarian tumours with significantly high levels in serous subtype. A decrease in Galectin-1 levels post-surgical intervention and after receiving chemotherapy was found. Galectin-1 levels evidently distinguished between normal, benign, malignant and metastatic cases as compared to CA125 levels. Galectin-1 demonstrated to be a better biomarker than CA125 according to the Receiver Operating Characteristic (ROC) curve analysis.

**Significance:**

The study emphasizes that serum Galectin-1 may serve as a better surrogate biomarker in Ovarian Cancer for early detection, discriminating between malignant and benign abdominal masses and monitoring the progression of the disease and response to treatment.

## Introduction

Ovarian carcinoma is a lethal gynaecological cancer with distinguishing biology at the molecular, cellular and clinical level [1, 2]. It is the eighth most common cause of death worldwide killing 140,000 women [3]. It has the worst prognosis and high mortality rate owing to the lack of proper screening, poor diagnosis and late detection of the disease, therefore termed a “silent killer” [4]. Epithelial ovarian tumours are most common, comprising of about 90% of ovarian tumours and are further categorized as: serous, mucinous, endometrioid and clear cell carcinomas [5]. Most of the women diagnosed at later stages have a 05 years survival rate of only 46% after getting standard treatment [6]. Initial treatment criteria for ovarian cancer include primary de-bulking surgery followed by paclitaxel/cisplatin adjuvant chemotherapy or neo-adjuvant chemotherapy (NACT) plus interval surgical cyto-reduction and adjuvant chemotherapy [7]. Cancer Antigen 125 (CA-125) is a useful biomarker for monitoring the progression of the disease yet lacks sensitivity as well as specificity as it increases in benign conditions like ovarian cysts, uterine fibroids and infections [8]. Thus there is a requirement for the identification of novel biomarkers for the screening of the disease.

There has been a breakthrough in the field of oncology with the discovery of galectins and since then they have taken a centre stage in cancer research due to their aberrant expression and immunosuppression in the tumours [9]. They are small molecular weight, soluble, glycan-binding proteins [10] that are implicated in regulating cell growth, cell adhesion, apoptosis, development and progression of tumours. In mammals, there are 15 members in the Galectin family out of which 13 have been identified as key proteins in the oncogenesis of different human cancer [11]. Galectin-1 is a prototype Galectin; first protein discovered in Galectin family [12]. It is a 14KDa β galactoside binding protein which participates in various biological functions like tissue development, cell proliferation, pre mRNA splicing and immunoregulation [13]. It is a homodimerizing pleiotropic protein whose function is dependent on its location as well as concentration. Galectin-1 shows intracellular as well as extracellular functions and is found both inside as well as outside the cell. Intracellularly it binds with various other proteins in sugar independent manner and extracellularly it interacts in sugar dependent manner with β galactoside glycol conjugates [14]. Galectin-1 has been detected in various malignancies like pancreatic cancer, hepatocellular cancer, prostate cancer, ovarian cancer and breast cancer. It is involved in various key processes of carcinogenesis like metastasis, angiogenesis and immunosuppression [15]. Serum Galectin-1 has been validated in various diseases like rheumatoid arthritis, high grade gliomas, neuroblastomas and head and neck squamous cell carcinomas as a potential biomarker and to monitor progression and response to treatment [16–19].

In this study we investigated serum levels of Galectin-1 in patients with ovarian tumours and its efficacy as a biomarker for diagnosis and monitoring progression and response to treatment.

## Material and methods

**Fig. 1:**
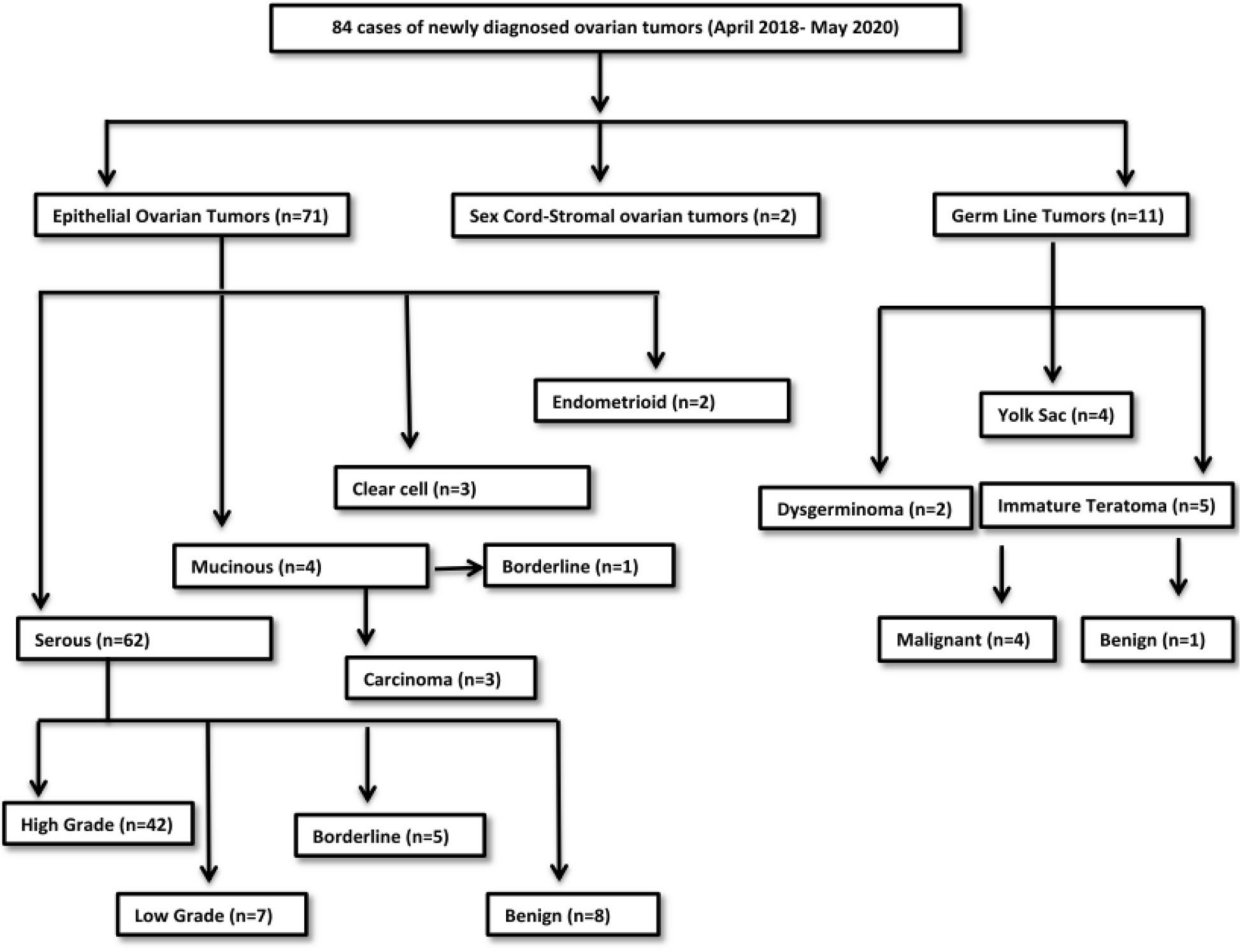
Flowchart of patient recruitment

This study comprised of 84 patients with newly diagnosed ovarian cancer and seeking treatment either cytoreduction surgery or conventional paclitaxel/carboplatin chemotherapy from Departments of Surgical and Medical Oncology respectively at Sheri-Kashmir Institute of Medical Sciences from April 2018 to May2020 (Fig. 1). Blood sample were collected from patients when diagnosed, after debulking surgery and post chemotherapy at different intervals. 20 healthy females were recruited as controls and samples were collected as desired. The study was approved by SKIMS IEC vide protocol no. 59/2018. Proper consent as required was taken from each patient and control enrolled in the study.

Blood samples were collected in a serum separator tube and allowed to clot at room temperature. The serum was separated by centrifugation, aliquoted and stored at -80°C till further use. Serum Galectin-1 was measured using human Galectin-1 Picokine ^TM^ ELISA kit from Boster Biological technology, USA according to the manufacturer’s guidelines. Serum CA-125 levels were measured by chemiluminescence technique using Beckman coulter reagents.

### Statistical analysis

Statistical analysis was performed by using Graph Pad Prism 5 software and all quantitative data were presented as mean ± S.D. Student’s t-test and one way ANOVA was used to analyze statistical significance between the variables studied. Correlation between Galectin-1 levels and CA-125 was determined by spearman’s rank correlation analysis. Receiver operator characteristic (ROC) curve was used to establish the cut-off values for Galectin-1 and CA125 serum levels with maximum sensitivity and specificity. The area under the curve was used to analyze the diagnostic accuracy of Galectin-1 and CA-125. A *p*-value <0.05 was considered statistically significant.

## Results

### Patient characteristics

An outline of patient characteristics is given below in the Table 1. 84.5% of the patients were diagnosed with epithelial ovarian cancer mostly with serous histology (Fig. 1) and at an advanced disease stage.

**Table 1:** Clinico- pathological characteristics of patients with ovarian tumours

Characteristics	*N*=84	
	No of patients	%age
Age (years)		
Mean age		46.66±15.42
Median		50
Range		14-70
>45	48	57.1
≤45	36	42.8
Menopause		
Pre Post	35 49	41.6 58.3
FIGO Stage		
I-II III-IV	27 46	32.1 54.7
Lymph nodes involved		
Yes No	28 56	33.3 66.6
Metastasis		
No Yes	41 43	48.8 51.1
Parity		
0 >1	20 64	23.8 76.1
Menstruation		
Normal Amenorrhea Dysmenorrhea Polymenorrhea	50 4 28 2	59.5 4.7 33.3 2.3

### Galectin-1 levels in patient with ovarian tumours

**Fig. 2:**
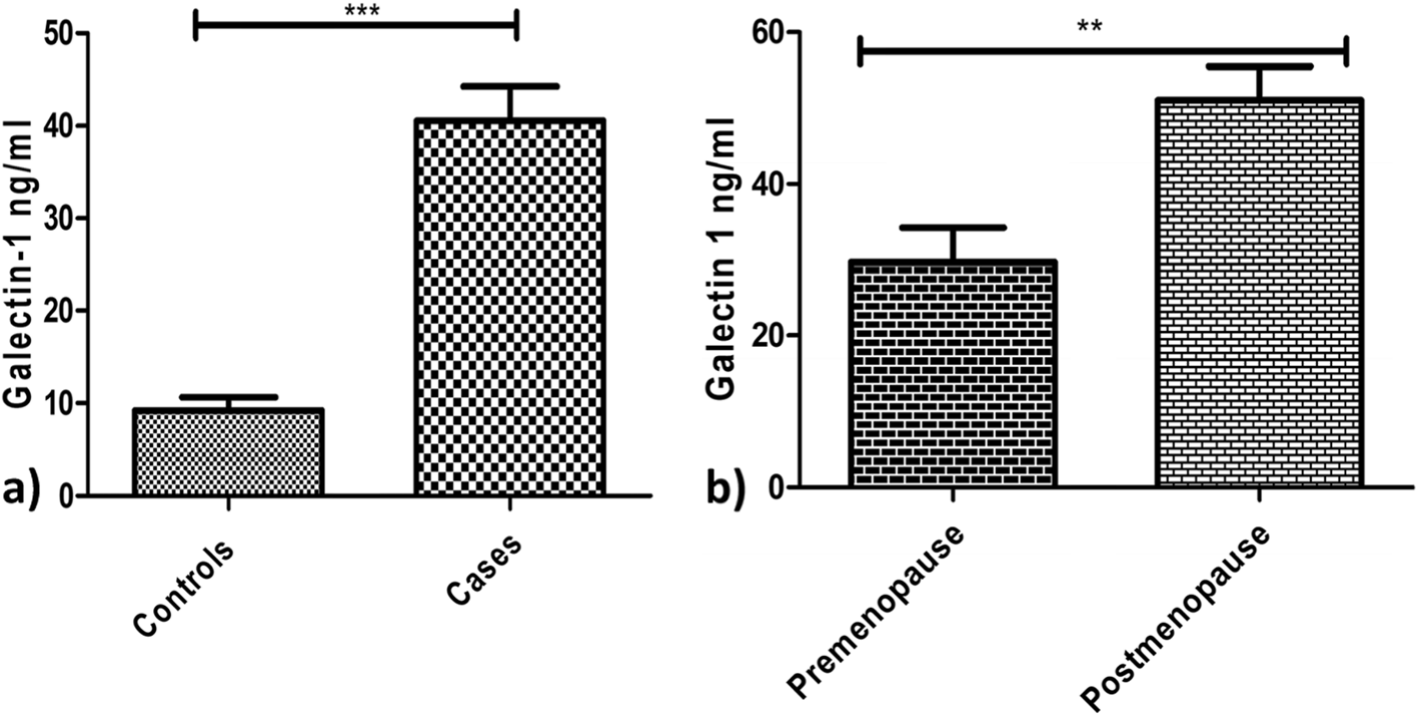
Serum Galectin -1 levels in a) healthy controls vs patients with Ovarian Tumours. b) Premenopausal patients vs postmenopausal patients

**Fig. 3:**
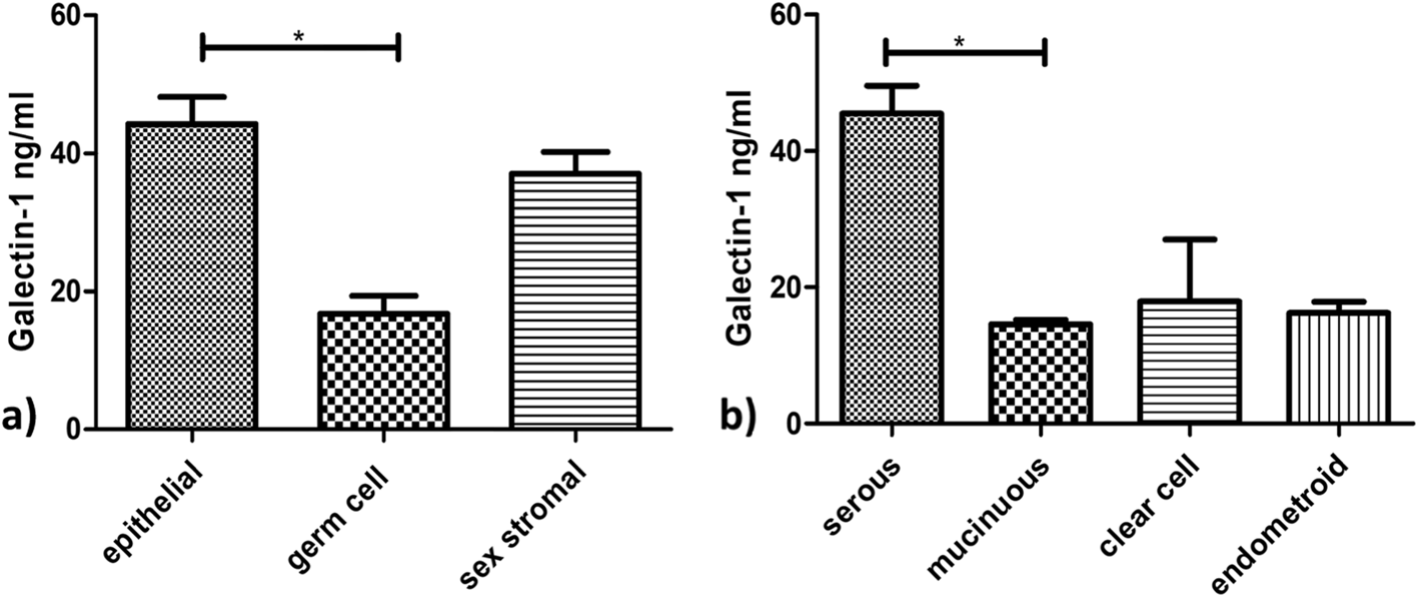
**a)** Serum Galectin-1 levels in ovarian tumour histotypes **b)** epithelial ovarian tumour subtypes

**Fig. 4:**
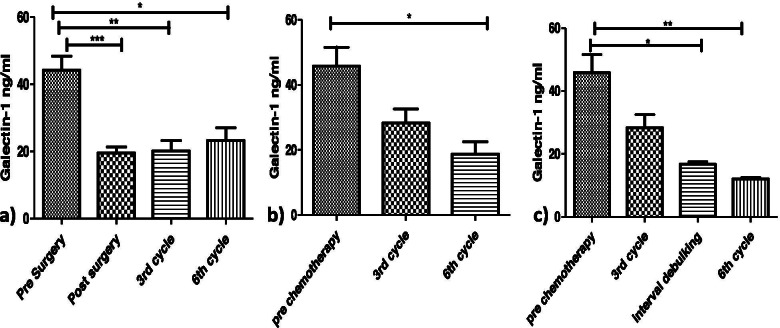
Serum Galectin -1 levels in OC patients (**a**) before and after surgery and adjuvant chemotherapy, (**b)** post neo adjuvant chemotherapy, (**c)** post NACT followed by interval cytoreduction and adjuvant chemotherapy

The serum levels of Galectin-1(40.57± 22.2 ng/ml) in patients with newly diagnosed ovarian tumours (*n*=84) were significantly higher than the levels (9.19± 4.61ng/ml) (*p*<0.0001) in healthy controls (*n*=20) (Fig. 2a). According to their menopausal status, in postmenopausal patients the Galectin-1 levels (51.02±19.95 ng/ml) were significantly higher than levels in premenopausal patients (29.66±18.13 ng/ml) (*p*<0.0021) (Fig. 2b). The Galectin 1 levels were analyzed further in patients categorized into the following groups:

- Galectin 1 levels in patients with different Ovarian Tumour Histotypes:
  Patients with epithelial ovarian tumours were significantly having high levels (44.22±22.06 ng/ml) than patients with germ cell ovarian tumours(16.71±6.51 ng/ml) **(***p*<0.0162) (Fig. 3a), however, no significant difference was found in serum levels of Galectin-1 in patients with sex stromal tumours (37.04±4.48 ng/ml) (*p*>0.05). Further, on comparing epithelial ovarian tumour subtypes with Galectin-1 levels, there was no significance found except between the patients with serous epithelial ovarian tumours (45.47±22.12 ng/ml) and mucinous ovarian tumours (14.55±1.32 ng/ml) (*p*<0.0071) (Fig. 3b).
- Galectin-1 levels in patients planned for primary debulking surgery
  The Galectin-1 levels in patients (*n*=70) who were planned for primary debulking surgery (44.20±21.45 ng/ml) showed a significant decrease in Galectin-1 levels post-surgery (19.50±9.03 ng/ml) (*p*< 0.0002). Out of 70 patients, 26 patients received 6 cycles of adjuvant paclitaxel carboplatin chemotherapy rest of the patients were having benign/borderline disease or were in the early stages of the disease, 2 patients died after surgery and hence were excluded from the study. After 6 cycles of adjuvant chemotherapy a significant decrease in Galectin-1 levels (23.29±9.98 ng/ml) was observed in patients than the levels pre-surgery (*p*< 0.05) (Fig. 4a). A non-significant increase in Galectin-1 levels was observed in these patients after receiving chemotherapy (p>0.05) as compared to levels after surgery.
- Galectin-1 levels in patients planned for NACT (Neo Adjuvant Chemotherapy)
  The patients (*n*=14) who were planned for NACT (45.83±15.31 ng/ml) received 6 cycles of chemotherapy and showed a significant decrease in the Galectin-1 levels post 6^th^ cycle (18.8± 6.42 ng/ml) **(***p*<0.0137) (Fig. 4b).
  The Galectin-1 levels of patients (*n* 6) who were scheduled for first 3 cycles of NACT and then interval cytoreduction (45.83±15.31ng/ml), showed a significant decrease in Galectin-1 levels post interval cytoreduction (16.7± 1.43ng/ml) (*p*<0.05) as well as after receiving 3 more cycles (12.15±0.72ng/ml) (*p*=0.0017) (Fig. 4c).

### Association of Galectin-1 and CA-125

**Fig. 5:**
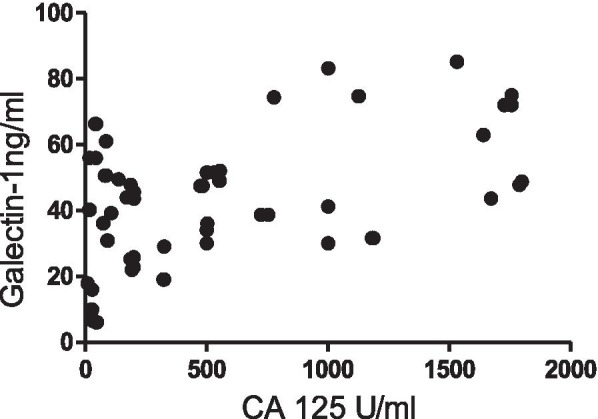
Correlation between Serum Galectin -1 levels with serum CA-125 levels in patients with Ovarian Tumours

**Fig. 6:**
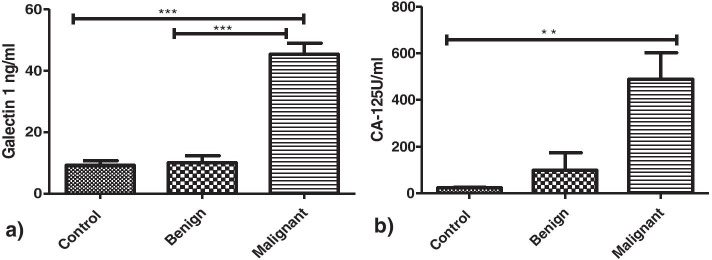
**a** Serum Gal-1 levels in controls vs benign vs malignant cases. **b** Serum CA-125 levels in controls vs benign vs malignant cases

**Fig. 7:**
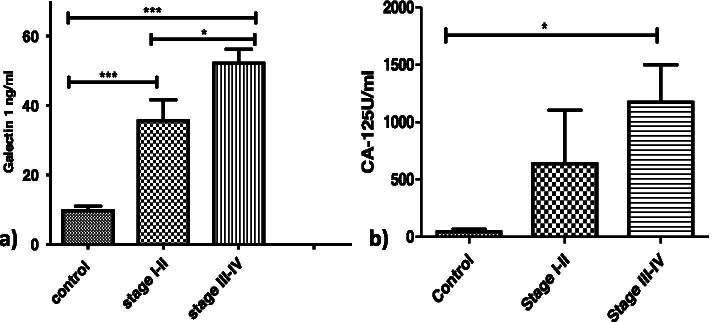
Serum levels of **a** Galectin -1 and **b** CA-125 levels in OC patients with different FIGO stages

**Fig. 8:**
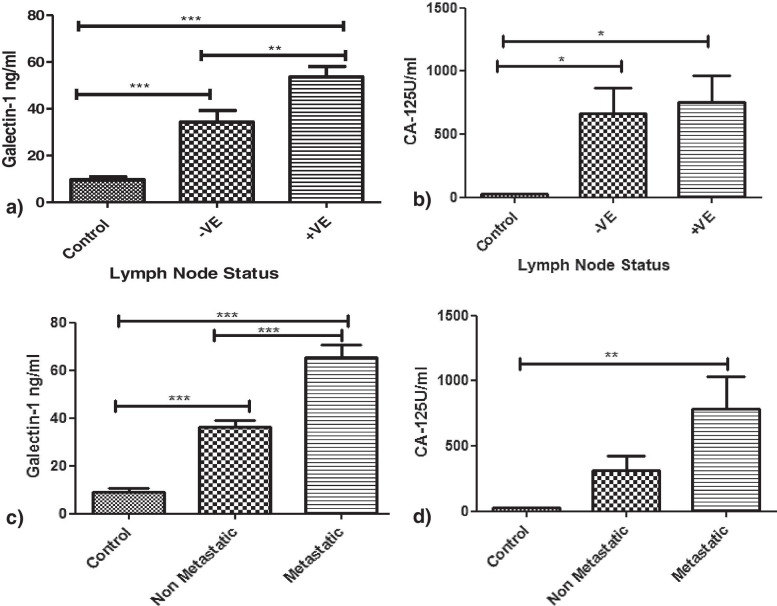
Serum Galectin -1 and CA125 levels in OC patients: **a** and **b** with (+ve) and without (-ve) lymph nodes involved. **c** And **d** with and without metastasis

Serum Galectin-1 levels from naïve patients positively correlated with their serum CA-125 levels (*n*=84, *r*=0.558, *p*<0.0001) (Fig. 5). Galectin-1 levels and CA-125 levels were analyzed in different patient groups as:

a) Ovarian tumour type: Galectin-1 levels in serum of patients with malignant ovarian tumours (45.35±19.88 ng/ml) were found to be higher significantly as compared to the controls (9.1±4.6 ng/ml) and benign ovarian tumours (10.0 ±5.29 ng/ml) (*p*<0.0001) (Fig. 6a). CA-125 levels were significantly higher in patients with malignancy (472.3±544.5U/ml) as compared to healthy controls (24.13±9.6U/ml) (*p*=0.0026) but there was no significant difference found between patients with malignant tumours and benign tumours (*p*>0.05) (Fig. 6b).

b) FIGO Stage: Serum Galectin-1 levels were significantly lower in controls (9.1±4.6 ng/ml) as compared to early stage patients (35.6± 21.8 ng/ml) (*p*=0.001) as well as late stage patients (52.22± 20.18 ng/ml) (*p*=0.001). A significant different in Galectin-1 levels was also observed between initial stages and later stages (*p*<0.05) (Fig. 7a). There was no significance in CA-125 levels at various stages of malignancy but a significant difference was found between higher stages (672±597.3U/ml) and controls (24.13±9.6U/ml)) (*p*=0.0365) (Fig. 7b).

c) Lymph Node Involved and Metastasis: Galectin-1 levels were significantly high in patients with lymph node involvement (53.6± 20.18ng/ml) than patients without lymph node involvement (34.33± 22.53ng/ml) (*p*=0.0021) (Fig. 8a). The CA-125 exhibited no significance between patients with and without lymph nodes involved (*p*>0.05) (Fig. 8b). Similarly Galectin-1 levels in patients with metastasis (52.3± 19.75ng/ml) were high than patients without metastasis (39.88±19.88ng/ml) (*p* =0.0001) (Fig. 8c). CA125 levels displayed no such significance between metastatic and non-metastatic patients (Fig. 8d).

### ROC curve analysis of serum Galectin-1and CA-125

**Fig. 9:**
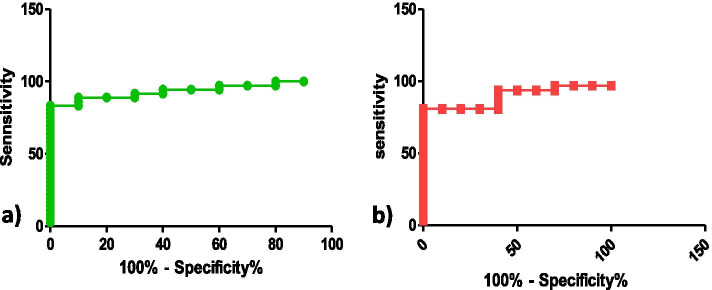
ROC curves depicting specificity and sensitivity of **a** Serum Gal-1 with AUC of 0.936 and **b** serum CA-125 with AUC of 0.89to distinguish between controls and OC patients

ROC curve analysis revealed an AUC of 0.936 (95% CI 0.8681 to 1.004**)** (*p*<0.0001) (Fig. 9a) for Galectin-1 and AUC of 0.89 (95% CI 0.7960 to 0.9911) (*p*< 0.0002) (Fig. 9b) for CA-125. The cut off value of >15.9ng/ml for serum Galectin-1 for the diagnosis of ovarian carcinoma has 88.89% sensitivity and 93% specificity whereas, for CA-125 cut off value of >36.5ng/ml has 80.65% sensitivity and 89% specificity Fig. 9.

## Discussion

Ovarian cancers are aggressive as they can progress from early stages to advanced stages within a year. The disease could have been curable if detected at early stages but remains undetected till advanced stages due to its late presentation [20]. Moreover the lack of effective screening and reliable biomarker has led to its high fatality [21]. CA-125 is currently used as biomarker for ovarian cancer but its specificity and sensitivity is limited. So far few serum markers like HE-4 have been evaluated but their efficacy as biomarkers for ovarian carcinomas still remains elusive [22, 23]. So in search of a better biomarker we have evaluated the levels of Galectin-1 in serum samples of patients diagnosed with ovarian tumors. Galectin-1 levels were higher in the serum of patients as compared to the healthy controls and same was reported by M. M. Abdel wahab et al. [24] and Chen et al. [25]. It is well know that menopausal status is a risk factor for ovarian cancer [26]. In our study it has been reported for the first time that postmenopausal women with ovarian tumours tend to have higher levels of serum Galectin-1 than premenopausal patients. In contrast Chen et al. [25] found no significance between serum Galectin-1 levels and menopausal status. Thus our study proposes high levels of galectin1 in postmenopausal women are an indication of a poor oncological outcome. Galectin-1 levels were high significantly in epithelial tumours as compared to germ cell tumours unlike Chen et al. who didn’t find any significant difference between various histologies of OC [25]. Galectin-1 levels were significantly high in serous tumours as reported by Chen et al. [25], Labrie et al. [27] and M. M. Abdelwahab et al. [24] than mucinous subtype as reported by Chen et al. [25] and histologically confirmed by Chetry et al [28]. According to the treatment paradigms, the naïve OC patients who were planned for primary debulking surgery showed a quite significant decrease in Galectin-1levels post debulking surgery which infers high serum levels of Galectin-1 are produced and secreted in serum by tumor and its tumor associated stroma [15]. Similar findings were reported by Chen et al [25] also but their sample size was small (10 patients) on the other hand our study was carried out on 57 patients (Fig. 1). After receiving 3 or 6 cycles of adjuvant chemotherapy a significant decrease was observed in Galectin-1 levels than the levels pre surgery. OC patients often show a high responsiveness to cisplatin chemotherapies at first but eventually develop platinum resistance. It has been reported that Galectin-1contributes to cisplatin resistance in EOC cells [13]. In our study, there was an increase in Galectin-1 levels with increasing cycles of chemotherapy but the results were insignificant. Thus there is a need to follow such patients for more cycles to corroborate these findings in developing chemo resistance. However, our study is the first to report Galectin-1 levels after more than 3 cycles of adjuvant chemotherapy. The group of patients who received NACT (NeoAdjuvant Chemotherapy) showed a significant decrease in Galectin-1 serum levels post 3^rd^ and 6^th^ cycles in contrast Coosmans et al [29] reported an increase in Galectin 1 levels post 3 cycle of chemotherapy. The patients who received 3 cycles of NACT followed by interval cytoreduction and then 3 cycles of adjuvant chemotherapy exhibited a significant decrease in serum Galectin-1 after interval cytoreduction and after 6^th^ cycle. This finding is being reported for the first time in such patients.

As CA-125 is used commonly as a biomarker for OC therefore we investigated its relationship with Galectin-1 serum levels and found a strong correlation between the two as was reported by Chen et al. [25] and M. M. Abdelwahab et al. [24]. In our study, Serum Galectin-1 levels were able to discriminate between benign and malignant tumours than serum CA-125 levels on contrary Chen et al. stated otherwise. The serum Galectin-1 levels differentiated between early and advanced stages of malignancy as reported by Chen et al. [25] and Abdelwahab et al. [24] and has been confirmed by functional studies by P Zhang et al. [13] whereas serum CA-125 levels could only differentiate advanced stage patients from healthy controls. Thus, supporting our hypothesis that unlike CA125, Galectin-1 correlates with clinical stage and can be helpful for the early detection of the disease. The serum Galectin-1 levels were distinguishable between the patients with and without lymph nodes involved but CA-125 levels couldn’t make such distinction. This has been another finding of our study. In our study OC patients with localized disease had low Galectin-1 levels as compared to patients with metastasis which are in tune with reports by Chen et al. [25] and Abdel wahab et al. [24]. On ROC curve analysis, Galectin-1 has a greater discriminative ability than CA-125 between OC patients and healthy controls with Galectin-1levels to be more sensitive and specific than CA-125 levels as was also reported by Abdelwahab et al. [24] and Labrie et al. [27].

## Conclusion

The study highlights the clinical significance of serum Galectin-1 as a suitable noninvasive biomarker for the early detection of the OC, monitoring response to treatment and an indicator of tumour metastasis and invasion.

## Data Availability

Contact Author for Data requests
